# Impact of Contemporary Redlining on Healthcare Disparities Among Patients with Gastrointestinal Cancer: A Mediation Analysis

**DOI:** 10.1245/s10434-024-16373-8

**Published:** 2024-11-01

**Authors:** Odysseas P. Chatzipanagiotou, Selamawit Woldesenbet, Muhammad Musaab Munir, Giovanni Catalano, Mujtaba Khalil, Zayed Rashid, Abdullah Altaf, Timothy M. Pawlik

**Affiliations:** 1 Department of Surgery, The Ohio State University Wexner Medical Center, Columbus, OH USA; 2 Department of Surgery, University of Verona, Verona, Italy

**Keywords:** Redlining, Residential segregation, Gastrointestinal cancer, Colorectal cancer, Hepatobiliary cancer, Mediation analysis, Oncological care, Appropriate treatment, Mortality

## Abstract

**Background:**

Historically, housing policies have perpetuated the marginalization and economic disinvestment of redlined neighborhoods. Residential segregation persists nowadays in the form of contemporary redlining, promoting healthcare disparities. The current study sought to assess the effect of redlining on oncological outcomes of patients with gastrointestinal cancer and identify mediators of the association.

**Methods:**

Patients with colorectal or hepatobiliary cancer were identified from the linked Surveillance, Epidemiology, and End Results (SEER)-Medicare database (2007–2019). The contemporary redlining index, a measure of mortgage lending bias, was assessed relative to disease stage at diagnosis, receipt of appropriate treatment, textbook outcome, and mortality. Mediation analysis was used to identify socioeconomic, structural, and clinical mediating factors.

**Results:**

Among 94,988 patients, 32.2% resided in high (*n* = 23,872) and highest (*n* = 6,791) redlining census tracts compared with 46.2% in neutral and 21.6% in low redlining tracts. The proportion of Black, Hispanic, and White patients experiencing high and highest redlining was 65.9%, 41.6%, and 27.9%, respectively. Highest redlining was associated with 18.2% higher odds of advanced disease at diagnosis, greater odds of not undergoing surgery for localized disease (adjusted odds ratio [aOR] 1.363, 95% confidence interval [CI] 1.219–1.524) or not receiving chemotherapy for advanced disease (aOR 1.385, 95% CI 1.216–1.577), and 26.7% lower odds of textbook outcome achievement. Mediation analysis for appropriate treatment quantified the proportion of the association driven by socioeconomic status, racial/ethnic minority status, racial/economic segregation, primary care shortage, and housing/transportation.

**Conclusions:**

Contemporary redlining contributed both directly, and via downstream factors, to disparities in oncological care and outcomes of patients with gastrointestinal cancer.

**Supplementary information:**

The online version of this article (10.1245/s10434-024-16373-8) contains supplementary material, which is available to authorized users.

Gastrointestinal (GI) malignancies represent a major public health challenge, totaling 26% of the global cancer incidence and more than one-third of all cancer-related deaths.^1^ Among GI cancers, colorectal, liver, and pancreatic cancer were responsible for approximately 3.1 million new cases worldwide in 2020 with the burden of pancreatic and colorectal cancer most pronounced in North America.^2^ In addition to patient and tumor-specific factors, potential geographic and social determinants of health contribute to access to care, as well as post-treatment outcomes.^3^ In particular, the current state of the housing sector reflects a long history of discriminatory housing policies, including redlining and exclusionary zoning, that have prevented minoritized individuals and low-income households from residing in healthy environments with high-quality public services and employment opportunities.^4^

The term “redlining” was coined when the Home Owners’ Loan Corporation (HOLC) produced color-coded maps that ranked neighborhoods based on the risk of mortgage lending, assigning the lowest grade “D” and coloring “red” neighborhoods with working-class and minority populations, especially Black and immigrant communities.^5,6^. Contemporary redlining persists with mortgage lending bias on the basis of property location, serving as a manifestation of structural racism and economic disinvestment that can have long-term effects on the health of patients with cancer.^7^ Analyzing the association between residential segregation and disparities in health outcomes requires accounting for the influence of multiple other factors, which form a complex ecosystem that can help to explain why certain patients present with more comorbidities or advanced disease, in addition to receiving different or inequitable care.^8^. To this end, utilization of mediation analysis may provide a statistical approach to deconstruct the factors involved in the complex relationship between redlining and outcomes of patients with GI cancer^9,10^ Understanding the impact of these contextual effects may help to identify targets for public health policies and multilevel interventions.^11^

To date, many studies have solely focused on mortality, have not investigated contemporary redlining indexing relative to healthcare disparities associated with residential segregation, and have failed to employ mediation analysis as a means to deconstruct the complex causal pathways linking redlining with cancer patient mortality.^12–16^ Therefore, the goal of the current study was to characterize the multifaceted role of redlining as a root cause of disparities in healthcare access, utilization, and clinical outcomes among patients with GI cancer. Demographic, socioeconomic, and structural factors that influence health inequities among patients residing in redlined neighborhoods were assessed. The utilization of mediation analysis allowed for the definition and quantification of the relative effect that different factors had along the causal healthcare pathway, as well as characterize how intermediate outcomes ultimately drive the effect of redlining on mortality among patients with GI cancer.

## Methods

### Study Population and Inclusion Criteria

**Fig. 1:**
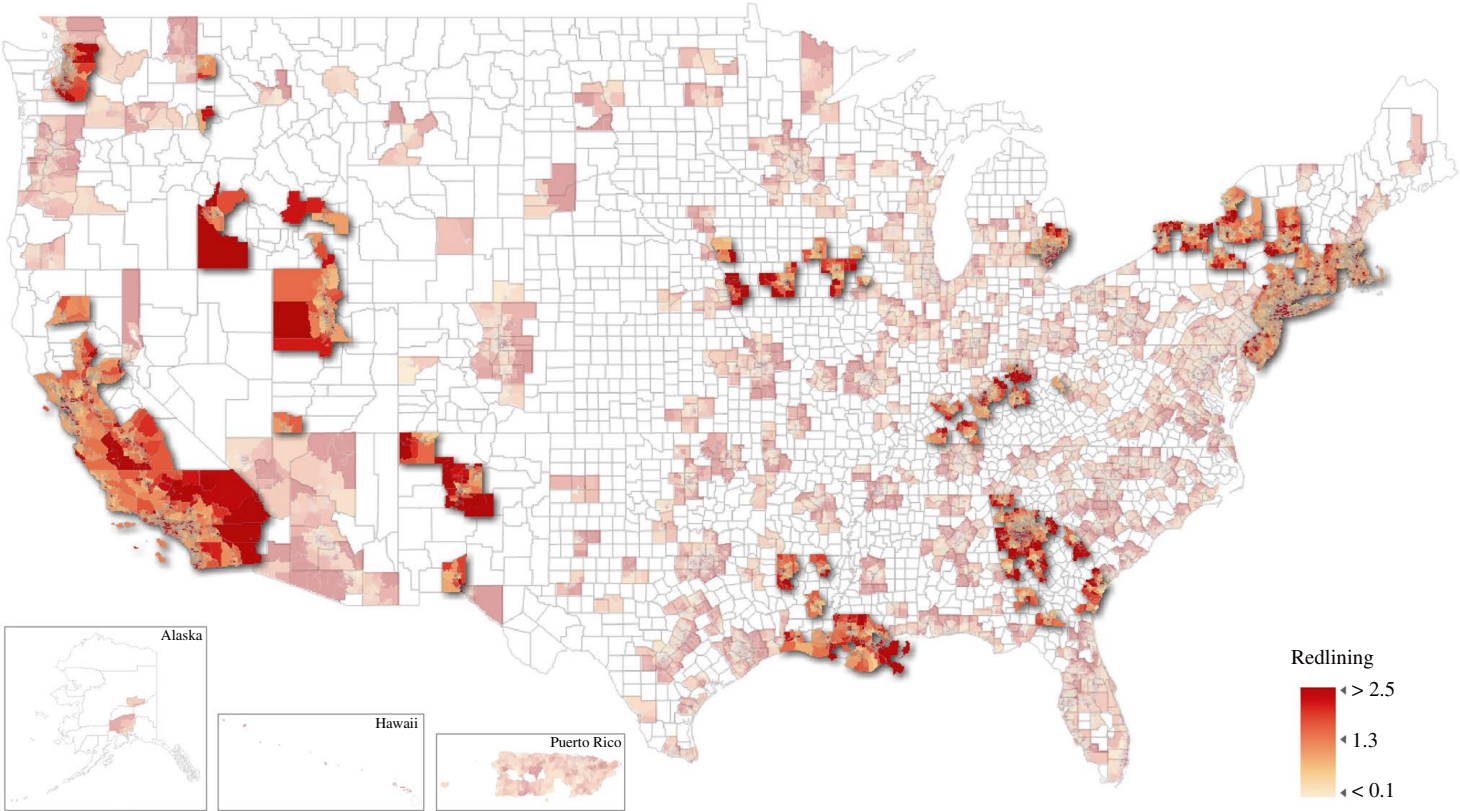
Census tract-level map illustrating the degree of redlining in metropolitan statistical areas within Surveillance, Epidemiology, and End Results registries

The Surveillance, Epidemiology, and End Results (SEER)-Medicare database was used to identify patients diagnosed with colorectal or hepatobiliary cancer, defined subsequently as “GI cancer.” The analysis was restricted only to hospitals within the SEER areas (Fig. 1). Patients were identified by using the International Classification Diseases of Oncology (ICD-O-3)/World Health Organization (WHO) 2008 SEER site recodes.^17^ The SEER program collects cancer incidence data from 18 registries and 15 states, accounting for approximately 30% of the U.S. population.^18^ The linkage with Medicare’s master enrollment file began in 1991 and is updated biannually, matching approximately 95% of individuals ≥65 years in the SEER files with each update.^19^

The initial cohort included 337,285 patients, excluding incidental diagnoses via autopsy. Initially, the cohort was restricted to patients enrolled in Medicare Parts A/B at least 12 months prior and after the GI cancer diagnosis. Further inclusion criteria were defined as diagnosis between 2007 and 2019, aged 66–90 years, known tumor stage (I–IV), not dying the same month as the diagnosis, and known census tract ID. Exclusion of patients outside of metropolitan statistical areas (MSAs) resulted in the final analytic cohort of 94,998 patients. The study was approved by the institutional review board of the Ohio State University.

### Variables and Outcomes of Interest

Demographic and clinicopathologic variables of interest included age, sex, race, Social Vulnerability Index (SVI), geographical region, year at diagnosis (i.e., 2007–2012, 2013–2019), cancer site, Charlson Comorbidity Index (CCI), American Joint Committee on Cancer (AJCC) stage, receipt of surgery and chemotherapy, achievement of textbook outcome, and vital status. Hospital procedural volume for each cancer type is defined as the total number of procedures performed in the hospital during the study period, divided by the number of years each procedure was performed within the same hospital. The CCI was used to assess comorbidities in the year prior to cancer diagnosis. Disease stage classification was defined based on the *AJCC Staging Manual*, using the sixth and seventh editions for patients diagnosed before and after 2010, respectively.^20,21^

Primary outcomes of interest included advanced disease at diagnosis, receipt of stage-appropriate treatment (e.g., surgery for localized disease, chemotherapy for advanced disease), and achievement of textbook outcome. Secondary outcomes included overall and cancer-specific mortality, in addition to examining interactions of redlining with age and race. Surgery for localized disease was defined as primary resection for stage I/II cancer, whereas chemotherapy for advanced disease was defined as systemic chemotherapy for stage III/IV cancer.^22^ Receipt of surgery was identified if recorded in Medicare Provider Analysis and Review (MEDPAR) files, while receipt of chemotherapy was identified if recorded in any of the SEER, MEDPAR, National Claims History, or outpatient files. SEER-available cause of death was used for cancer-specific mortality calculation. Textbook outcome was defined as a patient without any postoperative complications, as well as no extended length-of-stay, 90-day readmission, and 90-day mortality.^23,24^ Complications were captured and classified according to ICD9/10 codes. Length of stay was defined as days between date of surgery and date of discharge.^25^

### Main Exposure

The contemporary redlining index represents the odds ratio (OR) of mortgage application denial on the basis of property location compared with other properties within the same MSA.^7,12^ Home Mortgage Disclosure Act (HMDA) data for 389 MSAs (2010–2017) at the census tract level were used to develop the index and were made available online at the Medical College of Wisconsin website.^7,12^ A redlining index equal to 1.0 represented equal odds of mortgage application denial within and outside the census tract of interest.^12^ Odds ratios approximating 1.0 represented insignificant redlining. Based on equal distances from 1.0 on the reciprocal scale, a redlining index > 0.75 and ≤ 1.30 was considered neutral. In contrast, ORs ≤ 0.75 were categorized as low odds of mortgage denial; ORs > 1.30 and ≤ 2.50 were considered high odds of mortgage denial; and ORs ≥ 2.50 were regarded as highest probability of mortgage denial. The four categories were delineated as low (0–0.75), neutral (0.75–1.30), high (1.30–2.50), and highest (≥2.50) redlining. For mediation analysis, patients were stratified into no redlining (≤0.75) and redlining (>1.30) categories.

### Mediation Analysis

Mediation analysis was performed to identify the presence and quantify the contribution of factors that were affected by the exposure (redlining) and had an indirect effect on the outcomes of interest. Demographic and socioeconomic data for mediation analysis were obtained from the 2015 and 2020 five-year estimates of the American Community Survey, the Centers for Disease Control and Prevention/Agency for Toxic Substances and Disease Registry’s (CDC/ATSDR) SVI (2010, 2014, 2016, 2020), the CDC/ATSDR’s Environmental Justice Index, and the Health Resources and Services Administration’s Primary Care – Health Professional Shortage Area (HPSA) files.^11,26–32^ These publicly available datasets were linked to patients based on residential census tract (Tables S1 and S2, which break down the included variables**)**.

The SVI themes of interest were SV1 – Socioeconomic status, SVI3 – Racial/Ethnic Minority status, SVI4 – Housing & Transportation; in addition, the Environmental Justice Index’s Environmental Burden Module (EBM) was assessed.^26–30^ The HPSA file included designated areas and populations experiencing a shortage of primary care professionals. Census tracts with their HPSA status withdrawn before 2007 were excluded.^31^ The Index of Concentration of the Extremes (ICE) is a metric of “privilege” that was used to characterize an individual or a group of people as “privileged” versus others^11^. Index of Concentration of the Extremes ranged from −1 to +1, corresponding to a tract population being 100% Black individuals receiving less than US$25k/year versus 100% White individuals receiving more than US$125k/year, respectively.^32^

Generalized structural equation modeling (GSEM) was utilized for the assessment of the included variables as mediators of the effect between redlining and the outcome of interest in each model.^22,33^ Generalized structural equation modeling enables the simultaneous fitting of multiple models in a single analysis, with each outcome having each own distribution. Based on this, continuous outcomes were modeled by using Gaussian distribution. Poisson distribution and multinomial distribution were used for binary outcomes and categorical variables with >2 levels, respectively, whereas Weibull distribution was used for all-cause mortality.^22,23^

### Statistical Analysis

Summary statistics for each redlining index category were presented as median (interquartile range [IQR]) for continuous variables and as frequencies (proportion, %) for categorical variables. Comparisons were performed by using the *t*-test and the chi-square test for continuous and categorical variables, respectively. Multivariate logistic regression and Cox proportional hazards models were used to assess the association of redlining with outcomes of interest. The covariates included were age, sex, cancer site, region, as well as CCI (receipt of surgery, receipt of chemotherapy, textbook outcome), and hospital volume (textbook outcome). In a subanalysis, the interaction effect between redlining and age, as well as redlining and race were added to the multivariable model. All covariates had >95% completeness. Pair-wise deletion method was used for handling missing data. All tests were two-tailed, and a *p*-value < 0.05 was considered statistically significant. Levels of significance were adjusted for multiple comparisons using the Bonferroni correction method. All statistical analyses were performed by using STATA, version 17.0 (StataCorp, College Station, TX) and R version 4.3.2 (R Foundation for Statistical Computing, Vienna, Austria).

## Results

### Baseline Characteristics of Patient Cohort

A total of 94,998 patients were diagnosed with GI cancer between 2007 and 2019, including colon (*n* = 44,272; 46.6%), pancreatic (*n* = 23,326; 24.6%), hepatobiliary (*n* = 14,486; 15.2%), and rectal (*n* = 12,914; 13.6%) cancer. Median patient age was 77.0 years (IQR 71.0–83.0), and 53.7% of the cohort was female (*n* = 50.989). Among the entire cohort, 21.6% of individuals lived in low redlining census tracts, 46.2% resided in neutral redlining tracts, while 25.1% and 7.1% lived in high or the highest redlining tracts, respectively. Of note, 30.7% of Black patients lived in one of the highest redlining census tracts, which was markedly higher than the 4.8% of White, 8.6% of Hispanic, and 3.7% of other race/ethnicity populations in these tracts (*p* < 0.001). High and highest redlining neighborhoods were characterized by higher social vulnerability (high SVI: 37.5% vs. low SVI: 25.6%, *p* < 0.001) versus low redlining areas (high SVI: 18.6% vs. low SVI: 23.7%, *p* < 000.1; Table 1). Both highest (76.0 years, IQR 71.0–82.0) and high (77.0 years, IQR 71.0–83.0) redlining neighborhoods housed younger populations compared with low redlining communities (78.0 years, IQR 72.0–84.0) (*p* < 0.001; Table 2). Residing in a neighborhood with an increasing redlining score was also associated with higher likelihood to have CCI > 2 (27.8% vs. 33.1%), advanced AJCC stage (51.1% vs. 54.7%), treatment at a low case-volume hospital (24.7% vs. 33.8%), and lack of surgery for early-stage disease (37.8% vs. 41.3%) (all *p* < 0.001; Table 2).

**Table 1:** Cross-tabulation of redlining categories by age category, race, social vulnerability

	Race (p < 0.001)^a^				SVI (p < 0.001)^a^		
	White	Black	Hispanic	Other	Low	Moderate	High
Redlining							
Low	17,155 (23.9)	838 (10.4)	1,043 (13.9)	1,436 (18.7)	7,775 (23.7)	7,197 (22.1)	5,500 (18.6)
Neutral	34,585 (48.2)	1,916 (23.7)	3,347 (44.5)	4,015 (52.4)	16,674 (50.7)	14,177 (43.6)	13,012 (43.9)
High	16,597 (23.1)	2,853 (35.2)	2,487 (33.0)	1,935 (25.2)	7,096 (21.6)	8,541 (26.3)	8,235 (27.7)
Highest	3,375 (4.8)	2,487 (30.7)	647 (8.6)	282 (3.7)	1,309 (4.0)	2,592 (8.0)	2,890 (9.8)

Data are numbers with percentages in parentheses. SVI Social Vulnerability Index.^a^Pearson's chi-squared test

**Table 2:** Baseline characteristics of the cohort by redlining category

	Overall1	Low	Neutral	High	Highest	*p*^a^
No. patients	94,998	20,472 (21.6)	43,863 (46.2)	23,872 (25.1)	6,791(7.1)	< 0.001
Age (years)	77.0 (71.0 – 83.0)	78.0 (72.0–84.0)	77.0 (72.0–83.0)	77.0 (71.0–83.0)	76.0 (71.0–82.0)	< 0.001
*Sex*						0.060
Female	50,989 (53.7)	11,136 (54.4)	23,441 (53.4)	12,729 (53.3)	3,683 (54.2)	
Male	44,009 (46.3)	9,336 (45.6)	20,422 (46.6)	11,143 (46.7)	3,108 (45.8)	
*Minority status*						< 0.001
No	71,712 (75.5)	17,155 (83.8)	34,585 (78.8)	16,597 (69.5)	3,375 (49.7)	
Yes	23,286 (24.5)	3,317 (16.2)	9,278 (21.2)	7,275 (30.5)	3,416 (50.3)	
*Year at diagnosis*						< 0.001
2007-2012	46,919 (49.4)	9,848 (48.0)	21,429 (48.8)	12,033 (50.4)	3,609 (53.2)	
2013-2019	48,079 (50.6)	10,624 (52.0)	22,434 (51.2)	11,839 (49.6)	3,182 (46.8)	
*CCI*						< 0.001
≤2	67,601 (71.2)	14,790 (72.2)	31,560 (72.0)	16,711 (70.0)	4,540 (66.9)	
>2	27,397 (28.8)	5,682 (27.8)	12,303 (28.0)	7,161 (30.0)	2,251 (33.1)	
*Cancer site*						< 0.001
Colon	44,272 (46.6)	9,552 (46.7)	20,308 (46.3)	11,113(46.6)	3,299 (48.6)	
Rectum	12,914 (13.6)	2,715 (13.3)	5,907 (13.5)	3,386 (14.2)	906 (13.3)	
Pancreas	23,326 (24.6)	5,287 (25.8)	10,913 (24.9)	5,594 (23.4)	1,532 (22.6)	
Hepatobiliary	14,486 (15.2)	2,918 (14.2)	6,735 (15.3)	3,779 (15.8)	1,054 (15.5)	
*Region*						< 0.001
Northeast	5,674 (6.0)	1,805 (8.8)	1,525 (3.5)	1,969 (8.2)	375 (5.5)	
Midwest	8,013 (8.4)	1,497 (7.3)	2,881 (6.5)	2,167 (9.1)	1,486 (21.6)	
South	32,721 (34.4)	6,887 (33.7)	15,086 (34.4)	7,898 (33.1)	2,850 (42.0)	
West	48,590 (51.2)	10,283 (50.2)	24,371 (55.6)	11,838 (49.6)	2,098 (30.9)	
*AJCC stage*						< 0.001
I	20,940 (22.0)	4,564 (22.3)	9,660 (22.0)	5,319 (22.3)	1397 (20.6)	
II	24,966 (26.3)	5,447 (26.6)	11,666 (26.6)	6,175 (25.9)	1678 (24.7)	
III	20,151 (21.2)	4,286 (20.9)	9,394 (21.4)	5,028 (21.1)	1443 (21.2)	
IV	28,941 (30.5)	6,175 (30.2)	13,143 (30.0)	7,350 (30.8)	2273 (33.5)	
*Hospital volume*						< 0.001
Low	14,105 (27.9)	2,734 (24.7)	6,081 (25.8)	4,146 (33.1)	1.144 (33.8)	
Medium	17,903 (35.5)	3,752 (34.0)	8,738 (37.1)	4,435 (35.4)	978 (28.9)	
High	18,491 (36.6)	4,562 (41.3)	8,723 (37.1)	3,943 (31.5)	1,263 (31.5)	
*Surgery*						< 0.001
Yes	58,624 (61.7)	12735 (62.2)	27254 (62.1)	14647 (61.4)	3988 (58.7)	
No	36,374 (38.3)	7737 (37.8)	16609 (37.9)	9225 (38.6)	2803 (41.3)	
*Surgery (for localized)*						< 0.001
Yes	35,041 (76.3)	7,735 (77.3)	16,342 (76.6)	8,672 (75.5)	2,292 (74.5)	
No	10,865 (23.7)	2,276 (22.7)	4,984 (23.4)	2,822 (24.5)	783 (25.5)	
*Chemotherapy*						< 0.001
Yes	62,367 (65.7)	13,513 (66.0)	28,948 (66.0)	15,627 (65.5)	4,279 (63.0)	
No	32,631 (34.3)	6959 (34.0)	14,915 (34.0)	8,245 (34.5)	2,512 (37.0)	
*Chemotherapy (for advanced)*						< 0.001
Yes	35,186 (71.7)	7,570 (72.4)	16,324 (72.4)	8,786 (71.0)	2,506 (67.4)	
No	13,906 (28.3)	2,891 (27.6)	6,213 (27.6)	3,592 (29.0)	1,210 (32.6)	
*TO*						< 0.001
Yes	25,587 (50.7)	5,820 (52.7)	12,179 (51.7)	6,073 (48.5)	1,515 (44.8)	
No	24,912 (49.3)	5,228 (47.3)	11,363 (48.3)	6,451 (51.5)	1,870 (55.2)	
*Vital status*						< 0.001
Alive	24,771 (26.1)	5,526 (27.0)	11,847 (27.0)	5,920 (24.8)	1,478 (21.8)	
Deceased	70,227 (73.9)	14,946 (73.0)	32,016 (73.0)	17,952 (75.2)	5,313 (78.2)	

Data are medians with interquartile ranges in parentheses or numbers with percentages in parentheses unless otherwise indicated. AJCC American Joint Committee on Cancer; CCI Charlson Comorbidity Index; TO textbook outcome. ^a^t-test; Pearson’s chi-squared test

### Association of Contemporary Redlining with Outcomes of Interest

On multivariable analysis, after adjusting for relevant factors (i.e., age, sex, cancer site), residence in a high (ref: low; adjusted OR (aOR) 1.044, 95% confidence interval (CI) 1.004–1.085) and highest (ref: low; aOR 1.182, 95% CI 1.116–1.252) redlining area was associated with advanced disease at diagnosis. Of note, patients living in the highest redlining neighborhoods were associated with 36.3% greater odds of not undergoing surgery for localized disease (ref: low; high: aOR 1.146, 95% CI 1.120–1.173; highest: aOR 1.363, 95% CI 1.219–1.524) and 38.5% greater odds of not receiving chemotherapy for advanced disease (ref: low; high: aOR 1.140, 95% CI 1.046–1.242; highest: aOR 1.385, 95% CI 1.216–1.577). Among patients undergoing surgery, after adjusting for relevant patient- and hospital-level factors (i.e., age, sex, cancer site, CCI, hospital volume), odds of achieving a textbook outcome following a surgical procedure for a GI cancer were lowest among patients living in the highest relining areas (ref: low; aOR: 0.733, 95% CI 0.666–0.807) (Table 3).

**Table 3:** Association between redlining and various outcomes of interest

	Advanced disease at diagnosis^b^		No surgery (localized disease)^c^		No chemotherapy (advanced disease)^c^		Textbook outcome^d^	
Redlining	aOR (95% CI)	p	aOR (95% CI)	p	aOR (95% CI)	p	aOR (95% CI)	p
Low	ref		ref		ref		ref	
Neutral	1.017 (0.958–1.078)	0.5856	1.025 (0.995–1.056)	0.1068	1.020 (0.949–1.096)	0.5980	0.952 (0.915–0.990)	0.0147
High	1.044 (1.004–1.085)	0.0303	1.146 (1.120–1.173)	< 0.0001	1.140 (1.046–1.242)	0.0029	0.843 (0.819–0.868)	< 0.0001
Highest	1.182 (1.116–1.252)	< 0.0001	1.363 (1.219–1.524)	< 0.0001	1.385 (1.216–1.577)	< 0.0001	0.733 (0.666–0.807)	< 0.0001

^a^aOR adjusted odds ratio; CI confidence interval. ^b^Adjusted for age, sex, cancer site. ^c^Adjusted for age, sex, cancer site, Charlson comorbidity index. ^d^Adjusted for age, sex, cancer site, Charlson comorbidity index, hospital volume

After adjusting for relevant clinicopathologic factors (i.e., age, cancer site, year at diagnosis), redlining was also associated with higher hazards of all-cause (ref: low; high: adjusted hazards ratio (aHR) 1.095, 95% CI 1.067–1.123; highest: aHR 1.227, 95% CI 1.172–1.284) and cancer-specific (ref: low; high: aHR 1.069, 95% CI 1.036–1.103; highest: aHR 1.209, 95% CI 1.150–1.271) mortality (both *p* > 0.05; Table S3).

### Mediation Analysis

**Fig. 2:**
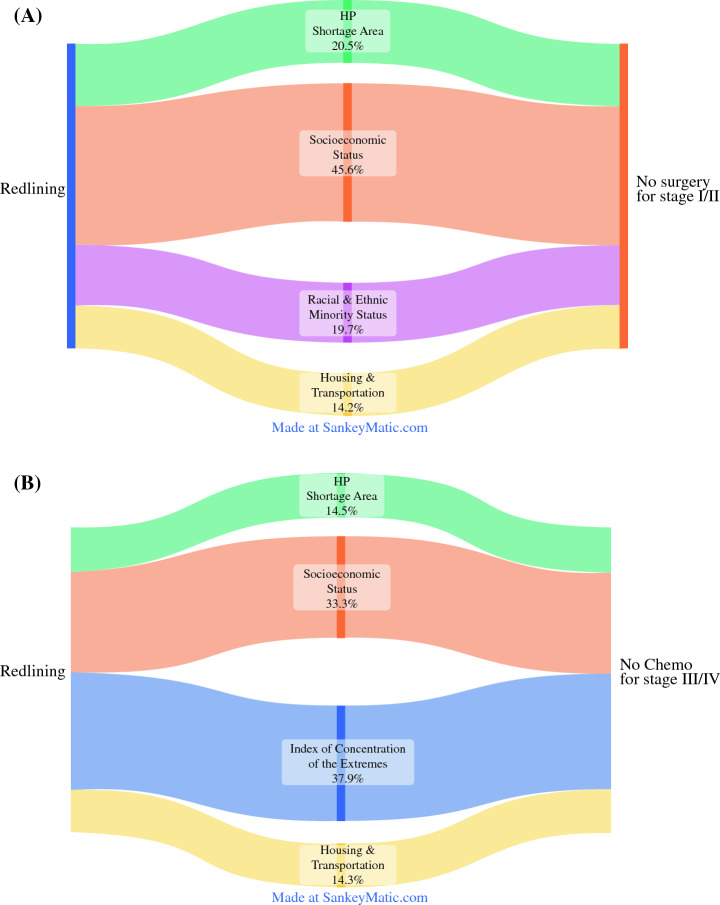
Sankey diagrams depicting the proportion mediated per mediating factor for (**A**) the association between redlining and no surgery for localized disease, and (**B**) the association between redlining and no chemotherapy for advanced disease. *HP* health professional

In assessing the direct and indirect effect between redlining and receipt of stage-appropriate treatment, the GSEM model demonstrated that socioeconomic status (relative risk [RR] 1.84, 95% CI 1.11–2.58) mediated 45.6% of the association between redlining and nonreceipt of surgery for localized disease. In addition, the HPSA status (RR 1.31, 95% CI 1.11–1.53) and racial/ethnic minority status (RR 1.30, 95% CI 1.11–1.49) contributed 20.5% and 19.7% of the effect of redlining on receipt surgery for patients residing in redlined tracts with localized GI cancer, respectively. Housing and transportation had a smaller effect (RR 1.21, 95% CI 1.11–2.58) accounting for 14.2% of the total association (all *p* < 0.05; Table 4; Fig. 2A). The association between redlining and non-receipt of chemotherapy for advanced disease was mediated 37.9% and 33.3% by ICE (RR 1.76, 95% CI 1.20–2.32) and socioeconomic status (RR 1.64, 95% CI 1.01–2.28), respectively. The HPSA status was responsible for 24% of the relative increase in the risk of patients residing in redlined areas not receiving chemotherapy for advanced disease (RR 1.24, 95% CI 1.08–1.40); 14.3% of the association was mediated by housing and transportation (RR 1.24, 95% CI 1.07–1.41) (all *p* < 0.05; Table 4; Fig. 2B). The total effect of redlining on receipt of stage-appropriate treatment was driven by mediators, whereas the indirect effect of EBM was insignificant in both GSEM models. Of note, the direct effect of redlining on all-cause mortality was 23.6%, whereas nonreceipt of appropriate cancer treatment, CCI, and advanced disease at diagnosis contributed the remaining 76.4% (Table 4; Fig. S1).

**Table 4:** Generalized structural equation modeling (GSEM) of mediation factors in GI cancer for outcomes of interests

Association		RR (95% CI)	Proportion mediated
Redlining ⇨ Nonreceipt of surgery for localized disease^a^	Direct effect	0.95 (0.80– 1.03)	–
	Indirect effect		
	Socioeconomic status	1.84 (1.11–2.58)	0.456
	Racial/ethnic minority status	1.30 (1.11–1.49)	0.197
	Housing and transportation	1.21 (1.01–1.42)	0.142
	HPSA	1.31 (1.11–1.53)	0.205
Redlining ⇨ Nonreceipt of chemotherapy for advanced disease^a^	Direct effect	0.98 (0.92–1.05)	–
	Indirect effect		
	Socioeconomic status	1.64 (1.01–2.28)	0.333
	ICE	1.76 (1.20–2.32)	0.379
	Housing and transportation	1.24 (1.07–1.41)	0.143
	HPSA	1.24 (1.08–1.40)	0.145
Redlining ⇨ Mortality^b^	Direct effect	1.05 (1.03–1.07)	0.236
	Indirect effect		
	Advanced disease at diagnosis	1.03 (1.01–1.05)	0.118
	Nonreceipt of appropriate treatment	1.11 (1.07–1.14)	0.484
	CCI	1.04 (1.02–1.05)	0.162

RR relative risk; CI confidence interval. ^a^Adjusted for age, sex, cancer site, Charlson comorbidity index. ^b^Adjusted for age, cancer site, year at diagnosis

### Interaction Terms of Redlining with Age and Race

Younger patients had greater hazards of all-cause mortality in both high (66–70: 14.7%; 71–75: 11.8%; 76–80: 9.0%; ≥80: 6.2%) and highest redlining census tracts (66–70: 39.0%; 71–75: 29.5%; 76–80: 20.6%; ≥80: 12.4%) versus patients in older age categories (all *p* < 0.05; Fig. S2). Of note, Black patients along the redlining index continuum had higher odds of presenting with advanced disease (highest: aOR 1.362, 95% CI 1.280–1.449l; high: aOR 1.245, 95% CI 1.148–1.350; neutral: aOR 1.279, 95% CI 1.157–1.414; low: aOR 1.362, 95% CI 1.213–1.530) compared with White patients living in low redlining neighborhoods (all *p* < 0.0001, Table S4).

## Discussion

Historically, residential segregation was supported by major economic institutions, maintained by federally backed housing policies, and strengthened by discriminatory practices of real estate agents and house sellers, ultimately restricting Black populations to the least desired neighborhoods.^34^ Existing literature on housing bias and oncological outcomes has, in the most part, focused on measures of ethnic density or composition.^35,36^ While some believe redlining is a historical phenomenon, there is evidence to suggest persistent housing discriminatory processes in relation to cancer outcomes.^12^ To this point, the contemporary redlining index represents mortgage lending bias on the basis of location alone and does not incorporate race/ethnicity of the applicant.^7^ However, residential segregation itself is interconnected with downstream socioeconomic, racial, and structural disparities, which have been proven to be independently associated with healthcare inequities.^37^ These mediating variables between redlining and outcomes of interest can be deconstructed by using mediation models, which aim to explain the underlying mechanisms that contribute to an observed association between the independent and dependent variables.^22^ The current study was important, because we assessed and identified contemporary redlining as an adverse independent predictor of advanced disease stage at diagnosis, receipt of stage-appropriate treatment, as well as short-term postoperative and long-term survival outcomes among patients with GI cancer. Additionally, the mediation analysis quantified the indirect effect that socioeconomic and racial/ethnic minority status, housing and transportation, racial/economic segregation, health professional shortages, and clinical factors had on the causal pathway relating redlining to receipt of appropriate treatment and all-cause mortality.

Present-day geographical distribution of disadvantaged communities is a consequence of historical policies that perpetuated social injustice and structural racism.^6,11,34^ In 1934, the Federal Housing Administration (FHA) attempted to improve housing sales by insuring private mortgages. To that end, color-coded maps from the HOLC were adopted, which ranked the perceived “stability” of neighborhoods. Black neighborhoods were usually rated low and colored in red—originating the term “redlining”.^5,6^ To date, high proportions of minority populations still reside in historically redlined neighborhoods, experiencing limited access to credit, employment, homeownership, and wealth accumulation.^5,10^ To this point, in 2019, 16% of Black and 11% of Hispanic applicants had their mortgage applications denied, while that was true for just 7% of White applicants.^4^ Along the same lines, 65.9% of Black and 41.6% of Hispanic patients in the current cohort resided in redlined tracts compared with just 27.9% of White patients (Table 1). A recent study by Miller-Kleinhenz et al. served to highlight the persistence of racial redlining among women receiving care for breast cancer.^38^ In summary, contemporary mortgage discrimination perpetuates race-based redlining that leads to inequalities in cancer care delivery.

Contemporary redlining is as a key driver of residential segregation that sustains health disparities on a personal, household, and neighborhood level.^7^ Distributive justice is “the right to equal treatment, that is, to the same distribution of goods and opportunities as anyone else has or is given.”^39^ In the context of redlining, distributive injustice entails exposure to disproportionate environmental hazards, limited access to healthcare, and poor infrastructure.^30,39,40^ Affordable and healthy food options are limited, communities are heavily targeted by tobacco and alcohol advertisements, and redlined populations have a higher likelihood of economic hardships, collectively experiencing acute and chronic cumulative stress.^34,37,40^ Of note, in the current study, increasing redlining correlated with increased burden among all social vulnerability subthemes, environmental hazards, primary care shortages, and racial/economic segregation (all *p* < 0.001; Table S2). Over the duration of almost a century, these circumstances have escalated the impact of redlining, insomuch that redlining was a strong independent adverse predictor of access to quality care and clinical outcomes, contributing to the disparities that patients may already face because of their race and socioeconomic status.^12–15^

Most contemporary literature on redlining has focused on its adverse effect on mortality, failing to examine the causal pathways that connect redlining with access to healthcare, healthcare resource utilization, postoperative course, and ultimately survival of oncological patients.^12^ Importantly, the current study demonstrated that although redlining directly impacts mortality (23.6%), the majority of the observed association was driven by indirect effects, including advanced disease stage at diagnosis (11.8%), nonreceipt of stage-appropriate treatment (48.4%), and CCI (16.2%). Focusing on these indirect associations, residing in high and highest redlining neighborhoods was associated with advanced disease stage at diagnosis (ref: low; highest: OR 1.182, 95% CI 1.116–1.252), as well as not receiving chemotherapy for advanced disease (ref: low; highest: OR 1.385, 95% CI 1.216–1.577) and not undergoing surgery for localized disease (ref: low; highest: OR 1.363, 95% CI 1.219–1.524). Collectively, patients with GI cancer living in redlined neighborhoods experienced inequalities throughout the care continuum and had worse oncologic outcomes. However, redlining was unlikely to be the sole factor responsible for the observed clinical outcomes.^35^ Other socioeconomic factors and neighborhood characteristics likely contribute to health disparities among patients with cancer.^10,41,42^ Therefore, mediation analysis was used to further analyze the association between redlining and nonreceipt of stage-appropriate treatment, demonstrating that socioeconomic status (45.6%), health professional shortages (20.5%), racial/ethnic minority status (19.7%), and housing and transportation (14.2%) contributed to not undergoing surgery for localized disease (Fig. 2A). Similarly, ICE as a proxy for racial/economic segregation (37.9%), socioeconomic status (33.3%), shortage of health professionals (14.5%), and housing and transportation (14.3%) indirectly drove the association between redlining and not receiving chemotherapy for advanced disease (Fig. 2B). These findings reveal the intricate web and dynamic interaction of socioeconomic characteristics inside neighborhoods, clinical factors and redlining, as well as highlight the potentially misleading causal inferences that can be introduced by treating possible mediators as confounders in statistical analyses.^10,11,43^

The observed association between redlining, advanced disease at diagnosis, and nonreceipt of stage-appropriate treatment can partly be attributed to the designation of lower-quality centers for disadvantaged neighborhoods, with limited specialty care options.^44^ Furthermore, redlined neighborhoods are more likely to face closures of healthcare-related facilities, pharmacy medication shortages, and low cancer screening rates.^11,40,45^ On a patient level, volatile schedules of low-wage workers and health facility shortages in redlined neighborhoods are additional obstacles that prohibit patients from allotting the time required for either surgery, hospitalization, and recovery, or chemotherapy appointments.^10,46,47^ As such, prior studies have linked historical redlining, expressed by HOLC grades, with advanced cancer stage at diagnosis, and lower likelihood (OR 0.67, 95% CI 0.54–0.82) of undergoing surgery at high-volume hospitals.^44,48,49^ Similarly, in the current study, increasing redlining index correlated with treatment at low-volume hospitals. Moreover, Black patients in highest redlining tracts had 36.8% higher odds of presenting with advanced GI cancer compared with White patients in low redlining tracts, in line with existing literature demonstrating that with increasing segregation, Black patients with GI cancer were more likely to both present with advanced disease and not receive stage-appropriate treatment.^10,22,35,36,43^

The results of the current study should be interpreted in light of certain limitations. Data linkages based on census tracts were utilized for the assessment of risk factors, possibly introducing limitations of generalizability and ecologic fallacy (i.e., making assumptions about individuals based on their surroundings).^22,50^ The redlining index was calculated by using inherently limited HMDA data, due to lack of information on credit score and employment status. Both the redlining index and mediating factors relied on data generated by various institutions on varying time scales, thus were not entirely reflective of the conditions experienced by patients^30^. The use of SEER-Medicare data for hospital procedural volume calculation has inherent limitations, because absolute volume cannot be ascertained owing to the exclusion of managed care beneficiaries, whose number varies per state, per year. In addition, the mediation model was inherently restricted to the factors that were assessed, potentially missing the effect of external factors. Future studies should aim to expand the mediation model and identify further mechanisms that contribute to the observed associations.

## Conclusions

Redlining was associated with disparities in equal access to healthcare, health resource utilization, and clinical outcomes among patients with GI cancer. A large proportion of inequities was mediated by clinical, demographic, socioeconomic, and structural factors faced by patients residing in redlined neighborhoods. In particular, redlining asserted its effect both directly and indirectly through a complex causal pathway that resulted in nonreceipt of appropriate treatment, as well as worse short- and long-term outcomes following surgical intervention for GI cancers. Rather than a historic remnant of discriminatory policies, contemporary redlining should be addressed to ensure equitable cancer care for all.

## Supplementary Information

Supplementary file 1 (DOCX 270 kb)

## Data Availability

The data for this study were obtained from the linked SEER-Medicare database. There are restrictions to the availability of this data, which is used under license for this study. Data can be accessed with permission from the National Cancer Institute and Center for Medicare and Medicaid Services.
