# Supplementary material for: Impact of Contemporary Redlining on Healthcare Disparities Among Patients with Gastrointestinal Cancer: A Mediation Analysis
Source: Ann Surg Oncol. 2024 Nov 1;32(2):1199–209. doi: 10.1245/s10434-024-16373-8 (PMC11698888; doi:10.1245/s10434-024-16373-8)
Supplement: Supplementary file 1 — (DOCX 270 kb) [file 10434_2024_16373_MOESM1_ESM.docx]

**Table S1.** Breakdown of Social Vulnerability Index (SVI) and Environmental Burden Module

| SVI 1 – Socioeconomic status | Poverty |
| --- | --- |
|  | Unemployment |
|  | Income (2010, 2014, 2016) |
|  | Housing Cost Burden (2020) |
|  | No High School Diploma |
|  | No health insurance |
| SVI 3 – Racial/Ethnic Minority Status | Race/Ethnicity – Minority status |
|  | English language proficiency (2010, 2014, 2016) |
| SVI 4 – Housing Type & Transportation | Multi-unit structures |
|  | Mobile Homes |
|  | Crowding |
|  | No Vehicle |
|  | Group Quarters |
| Environmental Burden Module | Air Pollution |
|  | Hazardous and Toxic Sites |
|  | Built Environment |
|  | Transportation Infrastructure |
|  | Water Pollution |

**Table S2.** Association of potential mediating factors with redlining

|  | **Overall^1^** | **No Redlining^1^** | **Redlining^1^** | **p-value^2^** |
| --- | --- | --- | --- | --- |
| ICE |  |  |  | <0.001 |
| High | 14,860 (33.3) | 1,646 (9.2) | 13,214 (49.5) |  |
| Moderate | 14,850 (33.3) | 5,171 (28.8) | 9,679 (36.3) |  |
| Low | 14,914 (33.4) | 11,117 (62.0) | 3,797 (14.2) |  |
| SVI 1 – Socioeconomic status |  |  |  | <0.001 |
| High | 14,816 (33.2) | 1,232 (6.9) | 13,584 (50.9) |  |
| Moderate | 14,893 (33.4) | 5,203 (29.0) | 9,690 (36.3) |  |
| Low | 14,909 (33.4) | 11,499 (64.1) | 3,410 (12.8) |  |
| SVI 3 - Racial & Ethnic Minority Status |  |  |  | <0.001 |
| High | 14,881 (33.3) | 2,880 (16.1) | 12.001 (45.0) |  |
| Moderate | 14,826 (33.2) | 6,985 (38.9) | 7,841 (29.4) |  |
| High | 14,920 (33.4) | 9,099 (45.0) | 6,851 (25.7) |  |
| SVI 4 - Housing Type & Transportation |  |  |  | <0.001 |
| High | 14,868 (33.3) | 3,140 (17.5) | 11,728 (43.9) |  |
| Moderate | 14,859 (33.3) | 5,432 (30.3) | 9,427 (35.3) |  |
| Low | 14,898 (33.4) | 9,362 (52.2) | 5,536 (20.7) |  |
| Health Professional  Shortage Area |  |  |  | <0.001 |
| Yes | 6,323 (12.4) | 751 (3.7) | 5,572 (18.2) |  |
| No | 44,812 (87.6) | 19,721 (96.3) | 25,091 (81.8) |  |
| Environmental Burden | 0.56 (0.31, 0.79) | 0.46 (0.24, 0.7) | 0.62 (0.37, 0.83) | <0.001 |
| *^1^*Median (IQR); n (%), *^2^*t-test; Pearson's Chi-squared test \| Abbreviations: ICE, Index of Concentration of the Extermes;; SVI, Social Vulnerability Index | | | | |

**Table S3.** Cox proportional hazards analysis of redlining with all-cause and cancer-specific mortality

|  | All-cause mortality^2^ | | Cancer-specific mortality^2^ | |
| --- | --- | --- | --- | --- |
| Redlining | aHR (95% CI)^1^ | p-value | aHR (95% CI)^1^ | p-value |
| Low | ref |  | ref |  |
| Neutral | 0.999 (0.965 – 1.034) | 0.9484 | 0.989 (0.960 – 1.019) | 0.4852 |
| High | 1.095 (1.067 – 1.123) | <0.0001 | 1.069 (1.036 – 1.103) | <0.0001 |
| Highest | 1.227 (1.172 – 1.284) | <0.0001 | 1.209 (1.150 – 1.271) | <0.0001 |
| *^1^*aHR = adjusted Hazards Ratio, CI = Confidence Interval; ^2^Adjusted for age, cancer site, year at diagnosis | | | | |

**Figure S1.** Sankey diagram depicting the proportion mediated per mediating factor for the association between redlining and all-cause mortality


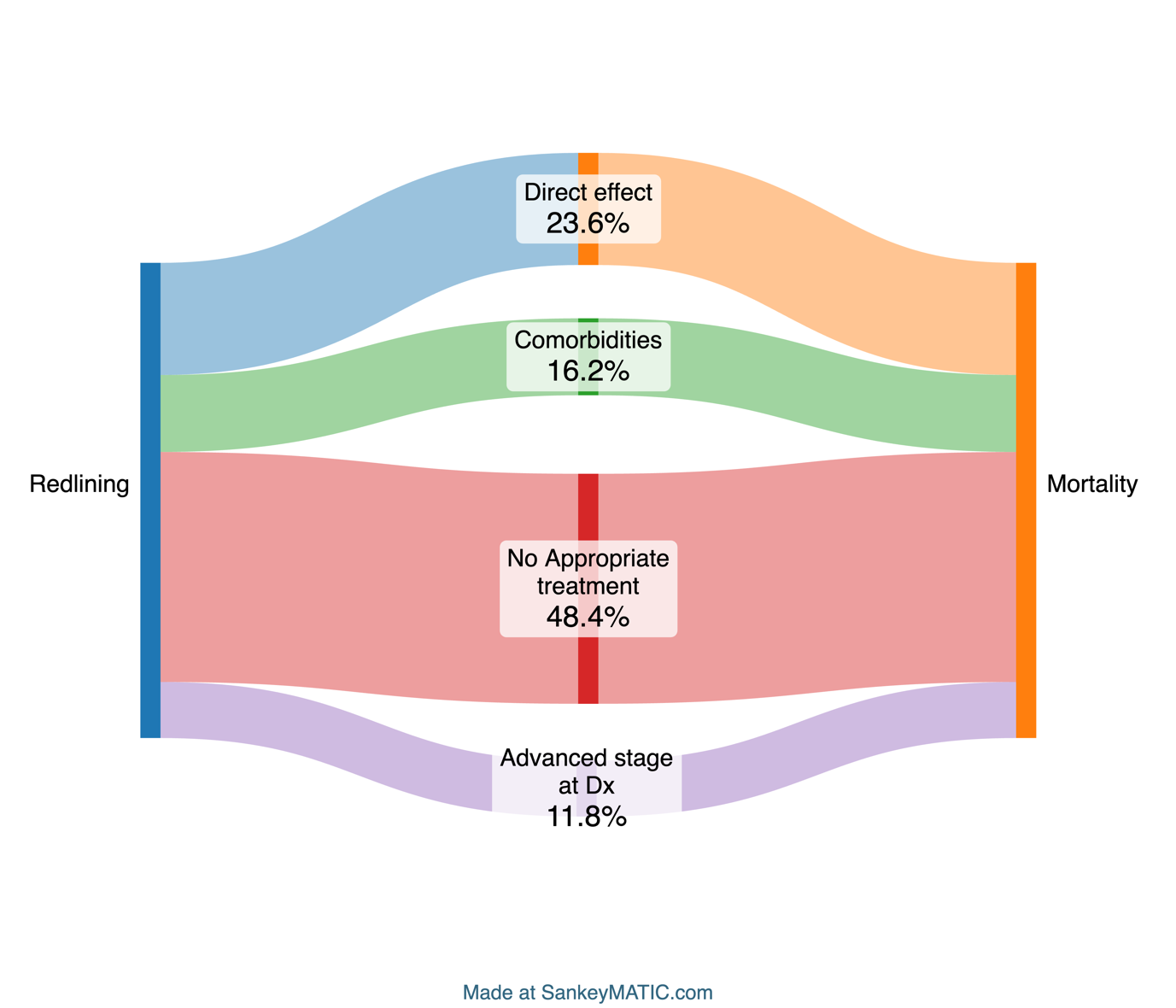


**Figure S2.** Relationship of all-cause mortality hazard ratios with increasing redlining index values, stratified by age category (66-70, 71-75, 76-80, >80); frequency distribution of the redlining index within the cohort.


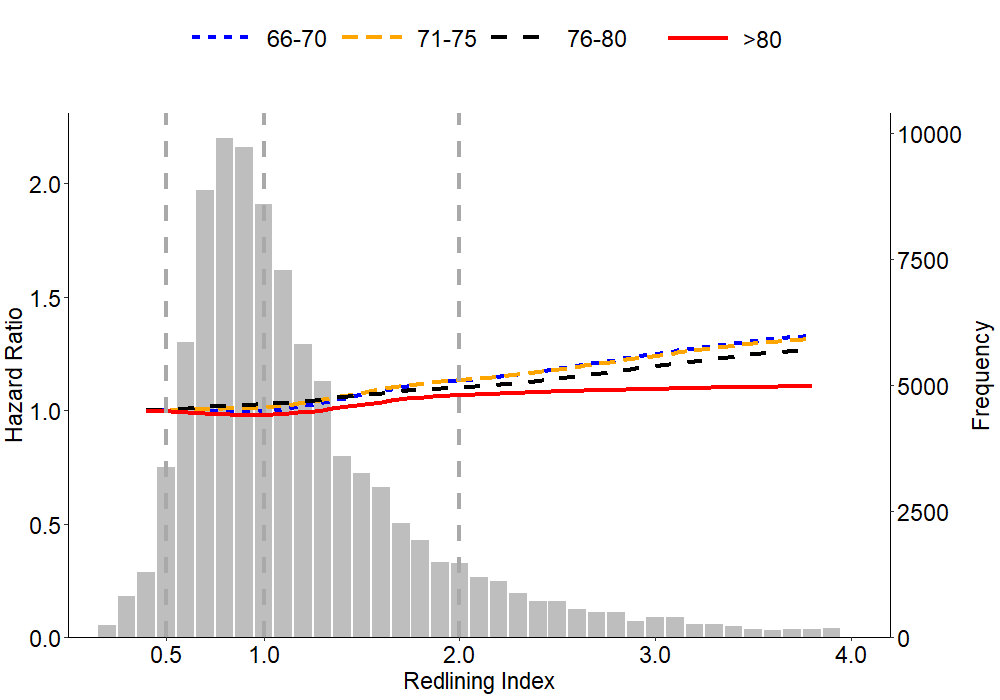


**Table S4.** Association of the interactions of (i) redlining & age with all-cause mortality, (ii) redling & race with advanced disease at diagnosis

|  | All-cause mortality^2^ | | | | Advanced disease^3^ |
| --- | --- | --- | --- | --- | --- |
|  | 66-70 | 71-75 | 76-80 | ≥ 80 | Black race  (ref: White; low redlining) |
| Redlining | aHR (95% CI)^1^ | aHR (95% CI) ^1^ | aHR (95% CI) ^1^ | aHR (95% CI) ^1^ | aOR (95% CI) ^1^ |
| Low | ref | ref | ref | ref | 1.362  (1.213 – 1.530) |
| Neutral | 1.017  (0.952 – 1.056) | 1.007  (0.971 – 1.045) | 0.998  (0.957 – 1.042) | 0.989  (0.939 – 1.042) | 1.279  (1.157 – 1.414) |
| High | 1.147  (1.091 – 1.207) | 1.118  (1.081 – 1.157) | 1.090  (1.055 – 1.126) | 1.062  (1.013 – 1.113) | 1.245  (1.148 – 1.350) |
| Highest | 1.390  (1.292 – 1.495) | 1.295  (1.220 – 1.374) | 1.206  (1.137 – 1.280) | 1.124  (1.046 – 1.209) | 1.362  (1.280 – 1.449) |
| *^1^*aOR = adjusted Odds Ratio, aHR = adjusted Hazards Ratio. CI = Confidence Interval; ^2^Adjusted for age, cancer site, year at diagnosis; ^3^Adjusted for age, sex, cancer site | | | | | |
